# A Rare Case of Adolescent Epiglottitis Secondary to Streptococcus dysgalactiae Septicemia

**DOI:** 10.7759/cureus.78671

**Published:** 2025-02-07

**Authors:** Kedar Tilak, Mohamed Aashiq Abdul Ghayum, Douglas Swanson, Rana El Feghaly

**Affiliations:** 1 Neonatology and Pediatric Infectious Diseases, Children's Mercy Kansas City, Kansas City, USA; 2 Pediatric Cardiology, Children's Mercy Kansas City, Kansas City, USA; 3 Pediatric Infectious Diseases, Children's Mercy Kansas City, Kansas City, USA

**Keywords:** acute epiglottitis, adolescent, adolescent and young adult, site of infection, streptococcus dysgalactiae

## Abstract

*Streptococcus dysgalactiae *(*S. dysgalactiae*)is a relatively uncommon pathogen in the pediatric populations, often overshadowed by *Streptococcus pyogenes *(*S. pyogenes*)in causing diseases such as cellulitis, severe seep tissue necrotizing infections, and streptococcal toxic shock syndrome. This case report presents the case of a 15-year-old recent immigrant male patient from Egypt who developed an extensive neck infection with complications from *S. dysgalactiae *septicemia. Initially misdiagnosed as viral pharyngitis, the patient was later admitted with high fevers, dysphagia, and progressive respiratory distress. Imaging revealed widespread inflammatory changes, including cellulitis, epiglottitis, and lymphadenopathy. Despite prompt antibiotic therapy, the patient required critical interventions, including intubation, inotropic support, followed by a tracheostomy. Blood cultures confirmed *S. dysgalactiae,* leading to tailored antibiotic therapy adjustments. This case underscores the pathogen’s potential to cause severe infections in pediatric patients, highlighting the need for early recognition and aggressive management. The clinical spectrum and burden of *S. dysgalactiae* remain underexplored, requiring further study to clarify its pathophysiology and infection patterns. Though rare in pediatric cases, particularly life-threatening septicemia, this organism should be considered alongside *S. pyogenes *in severe infections.

## Introduction

*Streptococcus dysgalactiae* (*S. dysgalactiae*) is a significant pathogen known to cause a range of diseases that are quite similar to those caused by *Streptococcus pyogenes* (*S. pyogenes*), including cellulitis, necrotizing soft tissue infections, and streptococcal toxic shock syndrome [1]. Research indicates that *S. dysgalactiae* can lead to both toxin-mediated and immune-mediated diseases [2]. The majority of the published literature on *S. dysgalactiae *infections focuses on the adult population, documenting cases such as pharyngitis, skin and soft tissue infections, and sepsis [3]. However, we found a report of *S. dysgalactiae* causing severe infection, as described in a pediatric patient with severe erythroderma, which was caused by *S. dysgalactiae* subsp. *equisimilis* (SDSE)* *[2]. In this report, we present a unique case involving a 15-year-old patient who developed epiglottitis as a complication arising from extensive neck infections secondary to *S. dysgalactiae* septicemia. This case highlights the potential severity of *S. dysgalactiae* infections and underscores the importance of recognizing and treating these infections promptly.

## Case presentation

**Figure 1:**
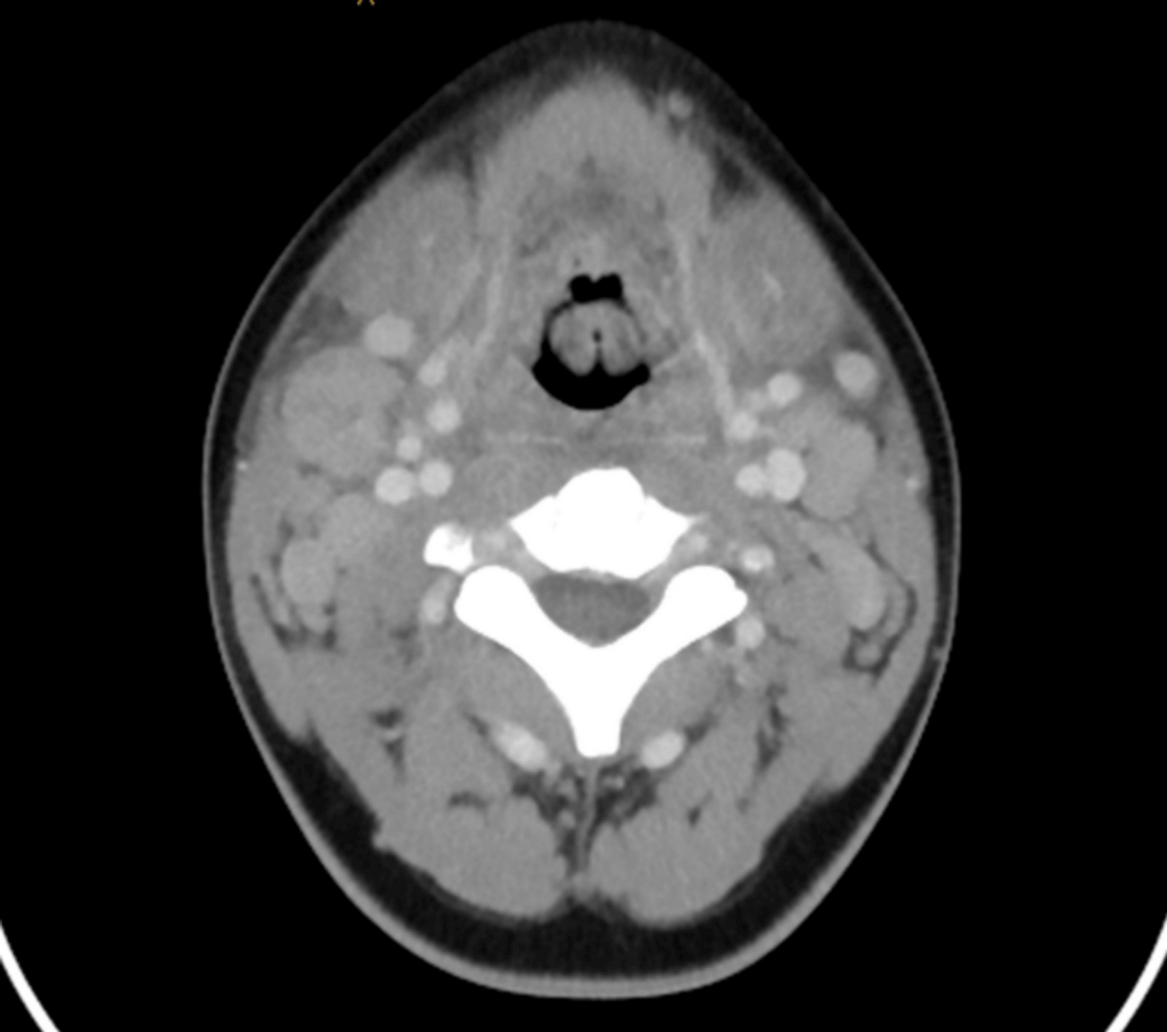
Initial CT obtained on day one showing epiglottis

Our patient was a previously healthy 15-year-old male who recently immigrated to the United States from Egypt around 80 days prior. He was in his usual healthy state until two days before presenting to our emergency department (ED) with complaints of fever and sore throat. He had been seen in local urgent care the previous day, where influenza and rapid streptococcal testing were negative, and he was discharged with a diagnosis of viral pharyngitis. Overnight, he continued to have high-grade fevers and developed dysphagia, so he presented to our ED for further evaluation. In the ED, his vital signs showed a temperature of 39.4°C, heart rate of 143 beats per minute (bpm), blood pressure of 123/58, respiratory rate of 20 respirations/minute, and oxygen saturation of 98% on room air. On his initial physical examination, he had mild neck swelling, but he rapidly deteriorated overnight with shortness of breath and a muffled voice. He required 2L oxygen support via nasal cannula to maintain his oxygen saturation above 95%. The significant labs from his initial blood work are summarized in Table 1. Blood cultures were obtained before the initiation of antibiotics. Due to concerns for retropharyngeal/peritonsillar abscess, a computed tomography (CT) scan of the neck was done, which showed extensive inflammatory changes of the neck with cellulitis, epiglottitis, prevertebral soft tissue swelling, diffuse soft tissue edema, mild upper airway narrowing, and bilateral cervical lymphadenopathy with tiny foci of necrosis without suppuration (Figure 1). He was started on cefepime, vancomycin, and metronidazole to facilitate broad coverage of potential pathogens. Two hours later, he suddenly developed biphasic stridor requiring otolaryngology consultation and immediate intubation. Following this, he was admitted to our pediatric intensive care unit (PICU), where he developed hypotension (64/35 mmHg) requiring significant ionotropic support. He continued to be persistently febrile. His echocardiogram showed poor left ventricular systolic function (26%). Infectious disease specialists were consulted. The initial blood culture grew *S. dysglactiae* after seven hours of incubation on the aerobic culture bottle. His antibiotics were de-escalated to ampicillin-sulbactam alone. However, his prolonged hospitalization and subsequent surgeries led to several antibiotic modifications to adequately treat the organisms growing on cultures with their respective susceptibilities. We have summarized this in Table 2. 

**Table 1:** Abnormal lab results from the patient's initial visit

Parameter	Obtained value	Reference range
White blood cell count	0.21 x 10³/mcL	4.0 - 11.0 x 10³/mcL
Absolute neutrophil count (ANC)	0	1.5 - 8.0 x 10³/mcL
C-reactive protein (CRP)	34.6 mg/dL	< 0.5 mg/dL
Hemoglobin	8.7 g/dL	13.8 - 17.2 g/dL (males)

**Table 2:** Summary of positive cultures, isolated organisms, and antibiotics during the hospital stay

Hospital Day	Site of culture	Organism	Antibiotics administered	Antibiotics modified to
1	Blood	Streptococcus dysgalactiae	Vancomycin, cefepime, metronidazole	Vancomycin, cefepime, and then narrowed to ampicillin-sulbactam
5	Neck abscess	Methicillin-resistant *Staphylococcus aureus* (MRSA), alpha-hemolytic streptococci, *Candida tropicalis*	Ampicillin- sulbactam	Vancomycin, clindamycin
	Nasal meatus right (Rt)/ left (Lt)	MRSA, alpha-hemolytic streptococci, *Candida tropicalis*		
	Nasal turbinate Lt	MRSA, alpha-hemolytic streptococci, *Candida tropicalis*		
	Nasal turbinate Rt	Cutibacterium acnes		
6	Tracheal	MRSA	Vancomycin, clindamycin	Clindamycin
7	Tracheal	MRSA	Clindamycin	Clindamycin
9	Neck abscess	*Streptococcus mitis*/ *Streptococcus oralis*, *Neisseria *species/ *Moraxella *species, MRSA	Clindamycin, meropenem, and linezolid	Clindamycin, ceftaroline, and micafungin
9	Neck abscess	*Actinomyces odontolyticus*, *Lactobacillus rhamnosus* (single colony), *Prevotella melaninogenica*	Clindamycin, ceftaroline, micafungin	Ceftaroline, micafungin, and metronidazole
13	Neck abscess	MRSA	Metronidazole, ceftaroline, and Micafungin	No changes

The hematology/oncology department was consulted to evaluate his low absolute neutrophil count (ANC). They attributed the acute pancytopenia to bone marrow suppression in the setting of critical disseminated bacterial illness. They requested polymerase chain reaction (PCR) to test for Epstein-Barr virus (EBV), cytomegalovirus (CMV), human herpesvirus 6 (HHV), adenovirus, and parvovirus B19, all of which were negative. They also tested his soluble interleukin-2 receptor (sIL-2R) and it was within the normal range. The allergy and immunology department was also consulted to evaluate for an underlying immunodeficiency disorder. He had a normal flow cytometry, oxidative burst panel, CH50, immunoglobulin M (IgM), and IgA levels and had an initial elevation in IgG levels, which later normalized. The HIV test was negative. A primary immunodeficiency gene panel was non-diagnostic. There were no pathogenic variants identified that were associated with immunodeficiency. He was a carrier for a single heterozygous pathogenic variant in Mediterranean fever (MEFV), which was consistent with carrier status for autosomal recessive familial MEFV. Several additional heterozygous variants of uncertain significance (VUS) were also noted, for which limited information is available. During his PICU admission, he developed intermittent ventricular tachycardia and atrial fibrillation, which required cardioversion and esmolol infusion followed by sotalol. Myocarditis was suspected, and a myocarditis viral panel PCR was sent, which came back negative. 

**Figure 2:**
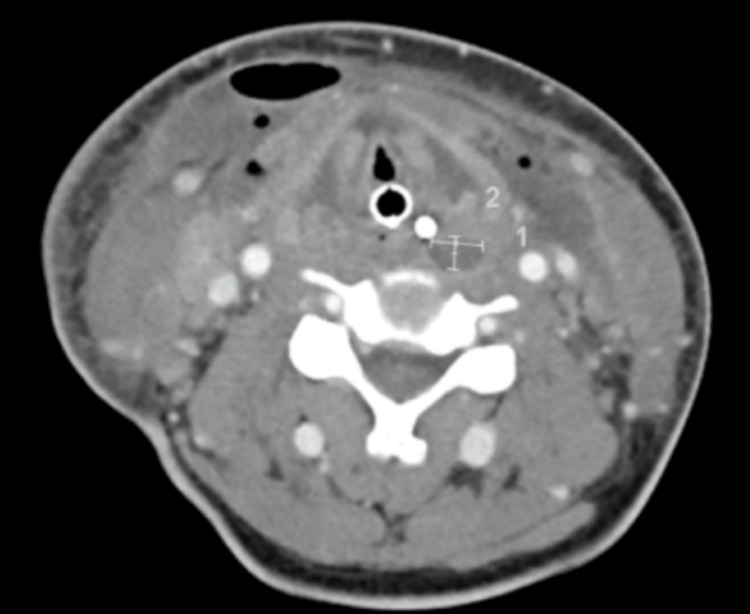
Repeat CT scan obtained on day five showing the neck abscess

On days five and six, he had excessive purulent secretions through his nasal cavity. A CT scan showed findings concerning for a new abscess in the neck. (Figure 2) He remained febrile with a low absolute neutrophil count (ANC). He was taken to the operating room (OR) for sinus biopsies to evaluate for acute invasive fungal disease (IFS). Pathology on biopsies showed rare, scattered Grocott methenamine silver (GMS) stain-positive structures resembling fungal yeast forms, but no fungal hyphae were seen. On hospital day seven, an ultrasound of the neck showed a new non-occlusive right subclavian vein thrombus and left internal jugular (IJ) vein narrowing. He had been receiving prophylactic heparin after the ENT procedure and was switched to therapeutic bivalirudin. The rest of the oncological evaluation, including a bone marrow biopsy, was negative, and his white blood cell (WBC) and ANC count eventually normalized. His neck continued to be erythematous and swollen. A repeat CT scan of the neck was obtained on day nine, which showed bilateral multiple neck abscesses with air involved, most notably in the right submandibular region and left level II-IV. This was concerning for necrotizing fasciitis, and he was taken to the OR immediately for transcervical right and left neck incision with abscess drainage and bilateral pharyngotomy with debridement of necrotic tissue.

**Figure 3:**
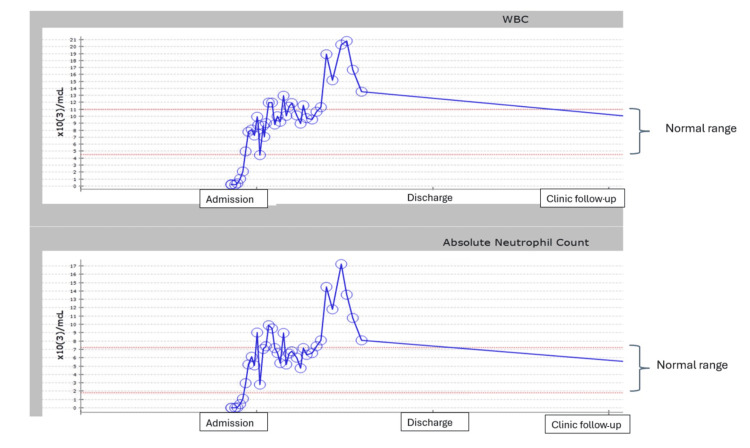
Total WBC count and ANC trend from admission until clinic follow up WBC: white blood cell; ANC: absolute neutrophil count

On day 11, he was taken to the OR again for repeat incision drainage and debridement of his right and left neck. After further debridement, his left neck had an exposed carotid sheath. He returned to the OR on day 15 for bilateral nasal endoscopy with maxillary sinus irrigation, bilateral neck exploration with mild debridement, and right vacuum-assisted closure (VAC). His neck wounds were showing significant improvement, and previous pharyngeal fistulae were nearly closed without evidence of necrotic tissue. On day 20, he underwent a tracheostomy and G-tube placement. Due to fevers and elevated C-reactive protein (CRP), he was transitioned to meropenem and linezolid on day 22, but as the patient did not have any further cultures that were positive, he was switched back to ceftaroline and metronidazole to complete a total seven-day course of therapy since his fever episode. On day 25, there were some new areas of necrotic tissue on the left neck with a fistula above the thyroid cartilage, and he returned to the OR for repair. The right neck wound was healing well. Neck and chest CT scans demonstrated no new fluid collection or free air in the neck but larger bilateral pleural effusions. Bilateral chest tubes were placed. He returned to the OR on alternate days for debridement and wound VAC changes. The open sites were closed after two weeks (hospital day 38). He had true vocal cord immobility on the right, which eventually improved. The left true vocal cord was mobile and normal in appearance. He was decannulated from the tracheostomy before discharge. A repeat ultrasound showed that the thrombus in his IJ had resolved, and the bivalirudin was discontinued. He was steadily weaned off ventilator support and was on a heat moisture exchanger (HME) at 21% during the day and a tracheostomy shield at night. He worked with speech therapy and used a Passy Muir valve (Passy-Muir, Inc., Irvine, CA) for speaking. His pleural effusions resolved status post chest tube placements. He received total parenteral nutrition (TPN) because he had multiple instances of being taken to the OR for debridement. Eventually, he was given enteral nutrition through a gastric feeding tube. Before discharge, he was stable with no supplemental oxygen support, and he was decannulated from his tracheostomy tube. We were trending his total white cell count and ANC throughout his hospital stay and even after discharge in the clinic, and Figure 3 shows a graph of the levels. He was discharged after a total inpatient hospital stay of 14 weeks.

## Discussion

Streptococci are gram-positive, nonmotile, catalase-negative cocci that occur in pairs or chains and are classified into three types based on the type of hemolysis seen on blood agar: alpha (α) hemolytic (incomplete hemolysis, appears green), beta (β)-hemolytic (clear due to complete lysis of red cells), and gamma (γ) hemolytic (no hemolysis) [4]. Lancefield group carbohydrate antigen is used to differentiate the species of β-hemolytic streptococci [5]. While *S. pyogenes* (group A) is the most common β-hemolytic streptococci, streptococci carrying serogroup C and G antigens, which include *S. dysgalactiae*, have been associated with a wide range of clinical infections in humans. *Streptococcus dysgalatiae* has two subspecies: *S. dysgalactiae* subspecies *dysgalactiae *(SDSD) and  SDSE (5). This organism traditionally used to be lumped together with group C *Streptococcus *but can be identified with the availability of molecular diagnostics like matrix-assisted laser desorption ionization time-of-flight mass spectrometry (MALDI-TOF). The common manifestations of the diseases caused by this organism include cellulitis, necrotizing soft tissue infections, and streptococcal toxic shock syndrome [1, 6, 7]. The organism adheres to the basement membrane and mediates colonization of the damaged epithelium. It can also activate the T-cell receptor molecules via exotoxins that increase T-cells and subsequently release cytokines [8].

There are very few case reports of *S. dysgalactiae* infections in the pediatric age group. A recent report by Okamura et al. [9] describes an 11-year-old girl with involuntary movements, clumsiness, and slurred speech who had group G *Streptococcus *identified from her throat culture. She was diagnosed with a presumption of secondary central nervous system (CNS) infection. However, there were no CNS cultures sent, and hence it is hard to attribute the CNS findings to the organism versus the throat cultures being positive due to colonization. Our patient did not have any neurological manifestations throughout his hospital stay. There are reports of patients presenting with a rash. Soler-Simon et al. [10] reported a 10-year-old girl who had a scarlatiniform rash, tonsillar hypertrophy, and cervical lymphadenopathy. She had a positive throat culture for SDSE. Besides this, we only found one other case in an adult 48-year-old male patient with similar cutaneous findings [11]. However, our patient never had a rash. Lopardo et al. [12] evaluated strain distribution, antibiotic resistance, and resistance mechanisms of *S. dysgalactiae*. In the study, they only found one neonate and two kids who had postsurgical infected wounds and cellulitis.

Kakuya et al. [13] evaluated 3,416 throat cultures and found 67 cultures with positive *S. dysgalactiae*. Most of these patients only presented with complaints of pharyngitis, fever, and lymphadenopathy. Seven of these 67 patients required hospitalizations, but none had serious complications. Our patient presented with initial pharyngitis, one of the common manifestations of a disease caused by *S. dysgalactiae*; he developed substantial complications with cellulitis, epiglottitis, and a necrotizing process, which are features that were not previously reported. Our patient had to undergo multiple debridement procedures with extensive bilateral pharyngotomies and had his epiglottis completely resected secondary to necrosis. His first blood cultures grew *S. dysgalactiae*, and he was in septic shock on the day of his cultures. Later in the course, he had multiple different bacteria grow from his subsequent surgical samples, but the original infection was driven by *S. dysgalactiae*. It is possible that some of the subsequent positive cultures were wound contaminants/colonization due to the fistula development. After a careful review of the existing cases reported and to the best of our knowledge, our patient had a unique clinical presentation with a complicated hospital course not described before. 

## Conclusions

The burden of *S. dysgalactiae* and the clinical spectrum of its diseases have not been fully studied and need to be further explored to gain a better understanding of the pathophysiology and infectious patterns associated with it. This unique case presentation demonstrated the range of systemic illness and severity of disease caused by *S. dysgalactiae*. A thorough immunological workup failed to demonstrate immune dysfunction, indicating the potential of this organism to cause severe systemic illness in immunocompetent children. Also, the organism is rarely recovered from infections in the pediatric population, especially life-threatening septicemia, and may need to be considered in situations where *S. pyogenes* is suspected.
